# Supplementary material for: SATB1 is a targetable modulator of JAK-STAT signaling and cytokines in human Treg and Tconv cells
Source: bioRxiv. 2026 Feb 16:2026.02.13.705474. Preprint. [Version 1] doi: 10.64898/2026.02.13.705474 (PMC13119340; doi:10.64898/2026.02.13.705474)
Supplement: Supplement 3 [file NIHPP2026.02.13.705474v1-supplement-3.pdf]

bioRxiv preprint doi: https://doi.org/10.64898/2026.02.13.705474; this version posted February 16, 2026. The copyright holder for this preprint (which was not certified by peer review) is the author/funder, who has granted bioRxiv a license to display the preprint in perpetuity. It is made available under aCC-BY 4.0 International license.

## Figure S1

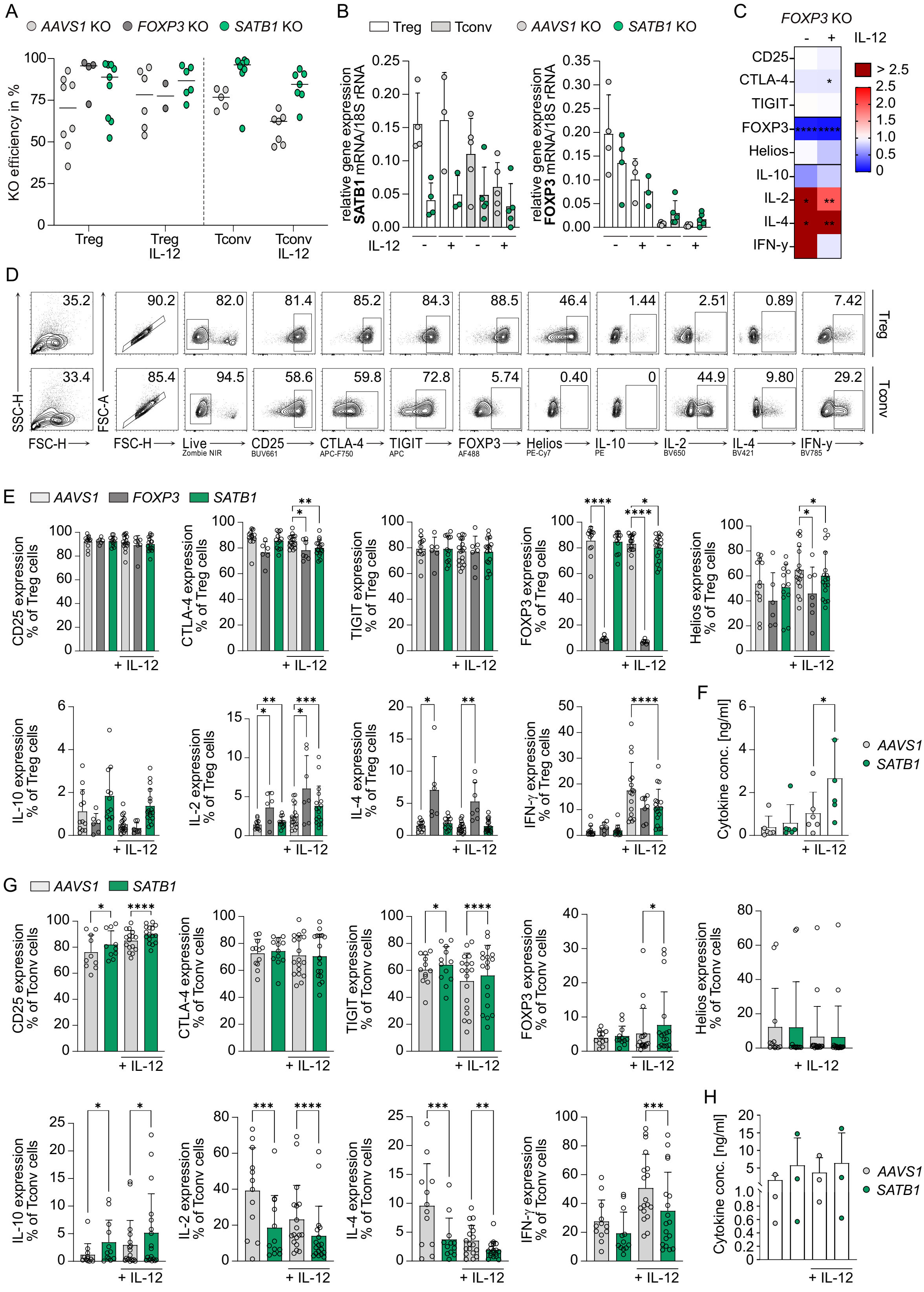

bioRxiv preprint doi: https://doi.org/10.64898/2026.02.13.705474; this version posted February 16, 2026. The copyright holder for this preprint (which was not certified by peer review) is the author/funder, who has granted bioRxiv a license to display the preprint in perpetuity. It is made available under aCC-BY 4.0 International license.

**Figure S1 - *SATB1* KO validation and overall phenotypic alterations in SATB1 ablated T cell subsets.** Human Treg and Tconv cells were isolated, expanded, activated and nucleofected with Cas9-RNPs targeting *AAVS1*, *FOXP3* and *SATB1*. The cells were cultured with or without the pro-inflammatory cytokine IL-12.

**(A)** Scatter dot plot displaying KO efficiencies with median. KO efficiencies were determined by amplicon NGS sequencing and TIDE analysis. n = 2-9.

**(B)** Bar graphs indicate mean of relative mRNA expression (ΔCt) of *SATB1* and *FOXP3* in *AAVS*1 and *SATB1* KO Treg cells normalized to 18S rRNA levels. RNA was isolated of FACS-sorted living cells. qPCR was performed in duplicates. n = 4-5.

**(C)** Flow cytometry analysis of canonical pro- and anti-inflammatory markers in *FOXP3* KO Treg cells stimulated with or without IL-12. Percentages of marker positive cells were normalized to the respective *AAVS1* KO Treg cells with or without IL-12 stimulation. n = 6-7, ratio paired t test.

**(D)** Flow cytometry gating strategy of *AAVS1* KO control Treg and Tconv cells without IL-12 conditioning.

**(E)** Bar graph plots quantifying flow cytometry marker expressions of *AAVS1*, *FOXP3* and *SATB1* KO Treg cells treated with or without IL-12. Data partially also shown in Fig. 1B & 1E. n = 6-17, paired t test.

**(F)** Extracellular IL-10 levels determined by LEGENDplex™ assay of control-treated *AAVS1* KO and *SATB1* KO Treg cells. n = 6, paired t test.

**(G)** Bar graph plots quantifying flow cytometry marker expressions of *AAVS1* and *SATB1* KO Tconv cells treated with or without IL-12. Data partially also shown in Fig. 1B &1G. n = 12-18, paired t test.

**(H)** Extracellular IL-10 levels determined by LEGENDplex™ assay of control-treated *AAVS1* KO and *SATB1* KO Tconv cells. n = 3, paired t test.

* p<0.05, ** p<0.01, *** p<0.001, **** p<0.0001.

bioRxiv preprint doi: https://doi.org/10.64898/2026.02.13.705474; this version posted February 16, 2026. The copyright holder for this preprint (which was not certified by peer review) is the author/funder, who has granted bioRxiv a license to display the preprint in perpetuity. It is made available under aCC-BY 4.0 International license.

## Figure S2

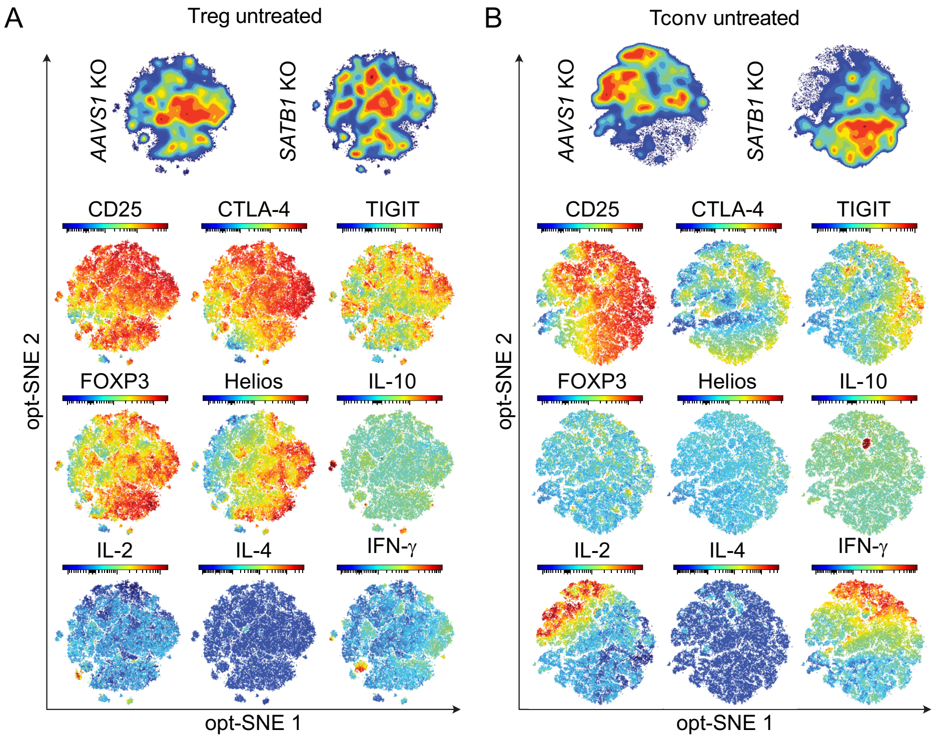


**Figure S2 - Integrated analysis of protein changes in *AAVS1* and *SATB1* KO Treg and Tconv cells based on flow cytometry.** opt-SNE density plot of untreated *AAVS1* KO and *SATB1* KO Treg **(A)** and Tconv cells **(B)**. Expression levels (MFI) of tested flow cytometry markers plotted on opt-SNE plot, n(Treg) = 18, n(Tconv) = 16.

bioRxiv preprint doi: https://doi.org/10.64898/2026.02.13.705474; this version posted February 16, 2026. The copyright holder for this preprint (which was not certified by peer review) is the author/funder, who has granted bioRxiv a license to display the preprint in perpetuity. It is made available under aCC-BY 4.0 International license.

## Figure S3

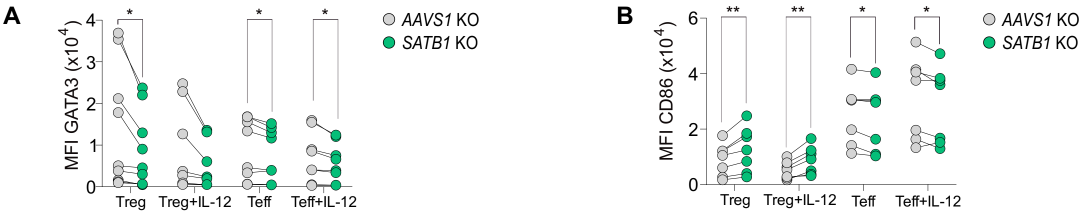


**Figure S3 - GATA3 and CD86 expression of *SATB1* KO and *AAVS1* control KO Treg and Tconv cells.**

**(A)** Mean fluorescence intensity (MFI) of GATA3 expression of *SATB1* KO and *AAVS1* control KO Treg and Tconv cells treated with or without IL-12. n = 6-9, paired t-test, * p<0.05.

**(B)** Mean fluorescence intensity (MFI) of CD86, and FOXP3 expression of *SATB1* KO and *AAVS1* control KO Treg and Tconv cells treated with or without IL-12. n = 8-9, paired t-test, * p<0.05, ** p<0.01, *** p<0.001.

bioRxiv preprint doi: https://doi.org/10.64898/2026.02.13.705474; this version posted February 16, 2026. The copyright holder for this preprint (which was not certified by peer review) is the author/funder, who has granted bioRxiv a license to display the preprint in perpetuity. It is made available under aCC-BY 4.0 International license.

# Figure S4

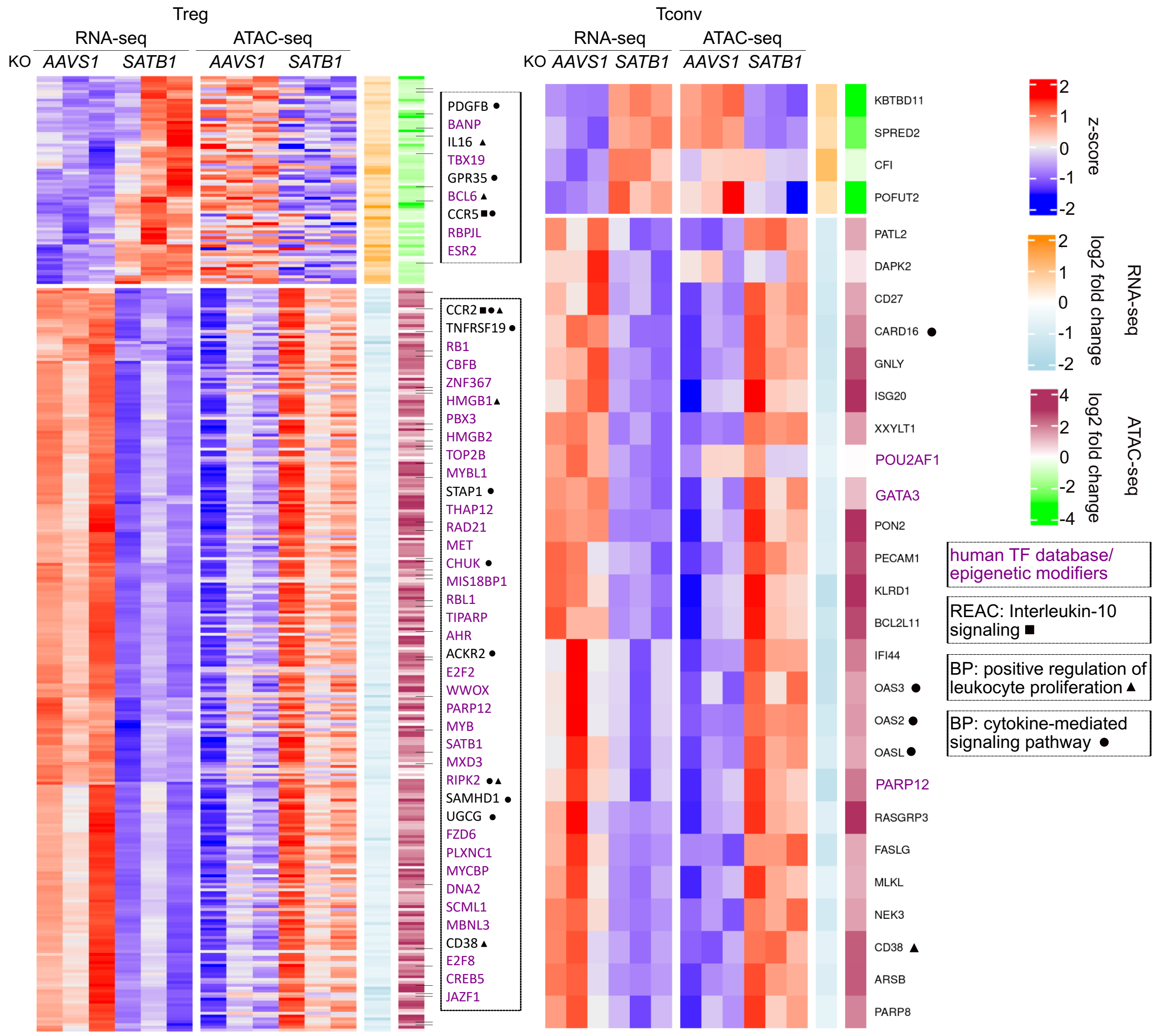


**Figure S4 - Genes differentially regulated on chromatin and transcription levels in *SATB1* KO Treg and Tconv cells.**
Heatmaps display z-scores of RNA- and ATAC-seq data of *SATB1* KO Treg and Tconv cells treated with IL-12. TFs differently regulated in RNA- and ATAC-seq data after *SATB1* KO are highlighted in purple. Genes associated with "Interleukin-10 signaling", "Positive regulation of leukocyte proliferation" or "cytokine-mediated signaling" are highlighted. REAC: Reactome; BP: biological pathway.
